# Selective Interaction of the Antimicrobial Peptide RKW with Bacterial Lipid Bilayers: A Biophysical Approach

**DOI:** 10.1021/acsomega.5c11601

**Published:** 2026-02-11

**Authors:** Alessandra Porritiello, Bruna Agrillo, Marta Gogliettino, Principia Dardano, Bruno Miranda, Adele Adamo, Emanuela Galatola, Marco Balestrieri, Gianna Palmieri

**Affiliations:** † 509065 Institute of Biosciences and BioResources (IBBR)-National Research Council (−CNR), Naples 80131, Italy; ‡ 96973 Institute of Applied Sciences and Intelligent Systems (ISASI)-National Research Council (−CNR), Naples 80131, Italy

## Abstract

Antimicrobial peptides (AMPs) have emerged as promising candidates for next-generation antibiotics due to their broad-spectrum activity, including efficacy against multidrug-resistant bacteria. However, their clinical application remains limited, primarily because of cytotoxicity toward host cells. A deeper understanding of AMP–membrane interactions, particularly through biophysical studies using model membrane systems, is essential for developing safe and effective AMP-based therapeutics. In this study, the interaction of a previously designed AMP, named RKW, with model lipid vesicles mimicking the lipid composition of both prokaryotic and eukaryotic cell membranes was investigated. RKW exhibited a strong preference for negatively charged bacterial membrane models, especially those representing Gram-negative bacteria, while showing minimal or no affinity for zwitterionic or eukaryotic-like membranes. These findings imply that electrostatic interactions are the primary driving force behind its membrane selectivity. Fluorescence spectroscopy and quenching experiments with acrylamide and lipophilic probes revealed that RKW localizes mainly at the membrane interface, likely adopting a parallel orientation relative to the bilayer surface. Furthermore, RKW induced substantial leakage of carboxyfluorescein from bacterial model membranes, indicating potent membrane permeabilisation. This mechanism was corroborated by dynamic light scattering (DLS) analyses, which provided additional evidence of peptide-induced membrane disruption. Collectively, this study elucidates the selective mechanism of action of RKW and underlines its potential as a targeted antimicrobial agent with reduced cytotoxicity toward eukaryotic cells. Toxicological assessments using the *Caenorhabditis elegans* in vivo model further supported its safety, showing no adverse effects on survival, reproduction, locomotion, or growth.

## Graphical abstract

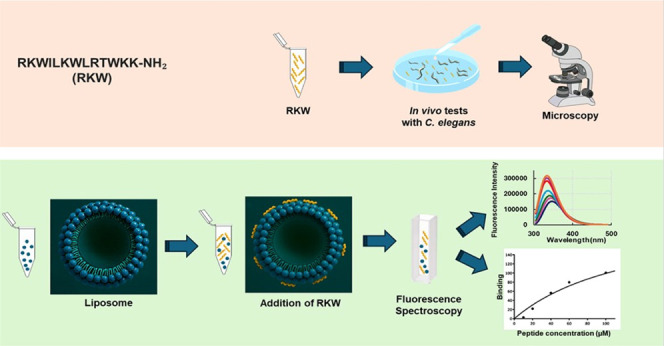

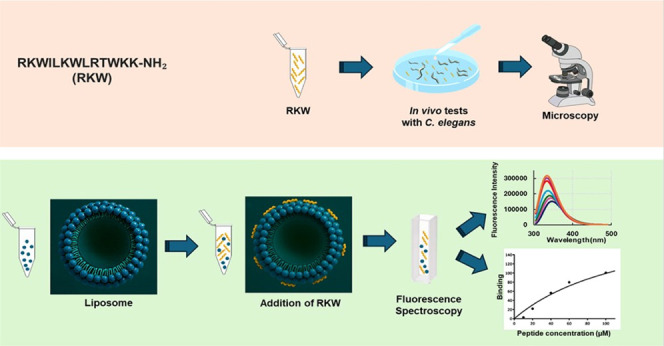

## 1 Introduction

The widespread use of conventional antibiotics has markedly reduced morbidity and mortality associated with bacterial infections. However, the emergence and rapid spread of drug-resistant bacteria have become a major global health concern, highlighting the urgent need for novel antimicrobial strategies.^1–3^ Antimicrobial peptides (AMPs) have emerged as promising alternatives due to their broad-spectrum activity against bacteria, fungi, parasites, and viruses, often at low micromolar concentrations.^1,4^ AMPs can be classified based on their amino acid sequence, net charge, hydrophobicity, length, and secondary structure. Structurally, they are divided into four major classes: β-sheet, α-helical, extended, and loop peptides.^5–7^ Many β-sheet AMPs possess disulfide-stabilized tertiary structures that confer enhanced stability.^2^ Despite their structural diversity, AMPs share conserved physicochemical properties, most notably cationicity and amphipathicity.^3,7^ Positively charged residues such as arginine and lysine facilitate electrostatic interactions with negatively charged bacterial membranes, while hydrophobic residues promote insertion into the lipid acyl chains, leading to membrane perturbation and disruption.

Unlike conventional antibiotics, which typically target intracellular components such as DNA or proteins, AMPs act primarily on bacterial membranes. This membrane-targeting mechanism lowers the likelihood of resistance development, as mutations that significantly alter membrane composition are often detrimental to cell viability.^8–10^ Bacterial membranes are rich in anionic lipids such as phosphatidylglycerol and cardiolipin, making them preferential targets for cationic AMPs.^7,11^ In contrast, mammalian membranes composed predominantly of phosphatidylcholine, cholesterol, and sphingomyelin are largely zwitterionic, resulting in weaker peptide binding and reduced cytotoxicity.

Moreover, the antimicrobial efficacy of AMPs is influenced not only by electrostatic interactions but also by various environmental and physicochemical factors, including temperature, pH, ionic strength, peptide-to-lipid ratio, and bilayer characteristics.^12^

Therefore, over the past decade, extensive research has focused on the rational design of novel AMPs with enhanced selectivity, structural stability, and antimicrobial potency.^1,8,13^ The primary objective of these efforts has been to elucidate the structural determinants that govern membrane interactions and selectivity, key factors for optimizing peptide design and therapeutic potential.

In this context, the attention was focused on RKW, a 13-residue cationic peptide that was previously rationally designed based on established structure–activity relationships of antimicrobial peptides.^14^ RKW was selected from a panel of short AMPs using a template-modified strategy guided by key physicochemical parameters governing peptide–membrane interactions. The design incorporated a Trp-based “WXXXW” motif, known to promote α-helical stabilization and amphipathic organization through Trp–Trp interactions. An integrated in silico analysis identified this peptide as a stable candidate with optimized physicochemical features, leading to its selection for biophysical and antimicrobial characterization. RKW adopts a canonical α-helical conformation in membrane-mimicking environments and exhibits remarkable structural stability under diverse conditions. Computational modeling indicates that RKW forms a well-defined amphipathic structure with distinct hydrophobic and cationic faces, a feature closely associated with its antimicrobial activity.^14^ Experimentally, RKW displays broad-spectrum antibacterial and antibiofilm activity against both Gram-positive and Gram-negative pathogens, including multidrug-resistant ESKAPE strains, as well as antifungal activity.^14^

Herein, the interactions of RKW with model membranes mimicking the lipid compositions of Gram-negative, Gram-positive, and mammalian cells were examined to elucidate the influence of lipid composition and peptide properties on membrane selectivity and permeabilization efficiency. A multidisciplinary biophysical approach combining fluorescence spectroscopy, fluorescence quenching, and dynamic light scattering (DLS) was employed to characterize peptide–membrane interactions. Our findings reveal that RKW predominantly localizes at the membrane interface, likely adopting a parallel orientation relative to the bilayer surface, and induces substantial carboxyfluorescein leakage from bacterial model membranes, indicative of strong membrane permeabilization.

Overall, this study provides detailed mechanistic insights into AMP–membrane interactions and underscores the potential of RKW as a model for the rational design of next-generation antimicrobial agents and biomaterials.

## 2 Materials and Methods

### 2.1 Synthesis of Peptide

The antimicrobial peptide RKW (RKWILKWLRTWKK-NH2) was purchased from GenScript Biotech (Leiden, Netherlands) at >95% purity. Analysis by mass spectrometry confirmed the identity of the peptide. It was stored as lyophilized powder at −20 °C, as recommended by the manufacturer. Before experimentations, fresh solutions in H_2_O or DMSO were prepared, briefly sonicated, and utilized as stock solutions in all analyses.

### 2.2 Lipids

The lipids 1-palmitoyl-2-oleoyl-*sn*-glycero-3-phosphoethanolamine (POPE), 1,2-dioleoyl-*sn*-glycero-3-phosphoethanolamine (DOPE), l-α-lysophosphatidylcholine (PC), phosphatidyl serine (PS), and 1′,3′-bis [1,2-dioleoyl-*sn*-glycero-3-phospho]-glycerol (Cardiolipin 18:1, CL), 5-NS (5-DOXYL-stearic acid), and 16-NS (16-DOXIL-stearic acid) were purchased from Avanti Polar Lipids (Alabaster, AL, USA). *N*-Acyl-d-sphingosine-1-phosphocholine (SM), and cholesterol (Chol) were purchased from Sigma-Aldrich (St. Louis, MO, United States), while 1-palmitoyl-2-oleoyl-*sn*-glycero-3-phosphatidylglycerol (POPG) was from Larodan (AB, Sweden).

The working buffer consisted of 4-(2-hydroxyethyl)­piperazine-1-ethanesulfonic acid (HEPES, Sigma-Aldrich) 10 mM and NaCl 100 mM (Sigma-Aldrich), pH 7.2.

### 2.3 Vesicle Preparation

Multilamellar vesicles (MLV), which were used in the binding assays and blue shift analyses, were prepared following the protocol outlined by Agrillo et al.^15^ Briefly, appropriate amounts of phospholipid stock solutions (10 mM) (Table 1) were dissolved in chloroform: methanol (2:1 v/v) and dried in a glass tube to form a lipid film under a stream of nitrogen, followed by high vacuum for at least 3 h to ensure complete evaporation of the organic solvent. Lipid samples were subsequently resuspended by vigorous vortexing in 10 mM HEPES, 100 mM NaCl, pH 7.2, to achieve a final concentration of 2 mM. The resulting MLVs were freshly prepared before each experiment.

**Table 1:** Lipid Composition of Eukaryotic and Prokaryotic Membranes^a^

	% mol							
membrane	PC	SM	PS	DOPE	POPE	POPG	CL	CHOL
eukaryotic^16^	25	12	8		25			30
zwitterionic	100							
*Salmonella typhimurium* ^GN,^ ^17^				78		18	4	
*Staphylococcus aureus* ^GP,^ ^18^						58	42	
*Pseudomonas aeruginosa* ^GN,^ ^19^					60	21	11	

a PC, phosphatidylcholine; SM, sphingomyelin; PS, phosphatidylserine; DOPE, dioleoyl-phosphatidylethanolamine; POPE, palmito-yl-oleoyl-phosphatidylethanolamine; POPG, palmitoyl-oleoyl-phosphatidylglycerol; CL, cardiolipin; CHOL, cholesterol. GN, Gram-negative bacteria; GP, Gram-positive bacteria.

Small unilamellar vesicles (SUVs) used in quenching and CD experiments were obtained by sonicating MLVs in a bath sonicator (Bandelin-SONOREX SUPER RK 100 H) for 15 min at room temperature with an ultrasonic frequency of 35 kHz.

### 2.4 Lipid Binding Assay

Peptide-phospholipid interactions were studied by monitoring changes in the Trp fluorescence emission spectra of the peptide upon the addition of MLVs, taking advantage of the presence of tryptophan residues in the RKW sequence. Fluorescence measurements were performed on a Shimadzu RF-6000 spectrofluorometer (Kyoto, Japan) at 25 °C and room temperature using a 1 cm path-length quartz cell (Hellma Analytics, Milan, Italy). Emission spectra were obtained between 300 and 450 nm using an excitation wavelength of 280 nm and slit widths of 5 nm.

Binding assays were performed by titrating the MLVs of varying lipid composition at a fixed concentration of 1.8 mM with increasing amounts of peptide solution ranging from 20 to 100 μM. The samples were vigorously vortexed and incubated at room temperature for 30 min. After incubation, the solutions were carefully transferred into polycarbonate centrifuge tubes (8 × 51 mm, Beckman Coulter, United States) and centrifuged at 60,000*g* (Beckman LE-80 Ultracentrifuge) for 1 h at 20 °C. Subsequently, the supernatant was removed, and the pellet was washed (two to three times) with the binding buffer (10 mM HEPES, NaCl 100 mM, pH 7.2) to remove any aggregated or precipitated peptide from the sample and resuspended in the same buffer containing sodium dodecyl sulfate (SDS, Thermo Fisher, Germany) at a final concentration of 1%. Fluorescence spectra were recorded for supernatant and pellet samples after 20 min of stirring at 900 rpm in a Vortex mixer (Labnet) at room temperature. The binding of the peptide RKW to model membranes was quantified by analyzing the distribution of peptide between the pellet (membrane-bound peptide) and supernatant (unbound peptide) fractions. Calibration curves were used to determine the concentration of RKW in each fraction, allowing for the calculation of the amount of peptide bound to the membranes. The calibration curves were generated by adding, immediately before the fluorescence measurements, known amounts of RKW into either the supernatant or pellet fractions of vesicles prepared in the absence of the peptide (see above).

For the blue-shift studies, peptide solutions (20 μM) in the working buffer were incubated for 30 min at 25 °C in the absence or presence of increasing concentrations of lipid vesicles (ranging from 0 to 1.8 mM).

### 2.5 Fluorescence Quenching of Trp Emission by Water-Soluble and Lipophilic Probes

Exposure of the Trp residue of RKW to the aqueous environment was evaluated by fluorescence quenching experiments using the water-soluble quencher acrylamide. Briefly, 2 μM of peptide solution in the absence and presence of SUVs at three different peptide/lipid (P/L) molar ratios (1:30, 1:50, 1:100) were titrated with increasing concentrations of acrylamide ranging from 0 to 200 mM. A 4.0 M stock solution of acrylamide was prepared in 10 mM HEPES and 100 mM NaCl, pH 7.2. Each spectrum was recorded after 30 min of incubation at 25 °C. To minimize the absorbance of the quencher, excitation of tryptophan was set at 280 nm.

The effect of the quencher on the fluorescence of the peptide was analyzed according to the Stern–Volmer equation

1 $$F_{0}/F=1+K_{sv}\left[Q\right]$$

where *F*_0_ and *F* represent the fluorescence intensity of the sample in the absence or presence of the quencher (*Q*), respectively, *K*_sv_ is the Stern–Volmer quenching constant, which is a measure of the accessibility of Trp to acrylamide, and [*Q*] is the concentration of the quencher.

Quenching studies with the lipophilic probes 5-NS or 16-NS were conducted by adding increasing concentrations of the quenchers (from 2.5 to 40 μM). Small aliquots of the stock solution (100 mM in ethanol) were added to samples of peptide (2 μM) in SUVs at three different P/L molar ratios (1:30, 1:50, 1:100) in 50 mM Tris–HCl containing 50 mM NaCl, pH 7.2, keeping the ethanol concentration below 2% (v/v) to avoid lipid bilayer perturbations.^20^ For every addition, a minimum of 30 min of incubation was allowed before measurement.

The fluorescence quenching data were analyzed according to the Stern–Volmer eq 1. All experiments were performed in duplicate, and the *K*_sv_ values were calculated from the slopes of the plots obtained by linear regression analysis.

### 2.6 Lipid Vesicle Leakage Assays

The ability of RKW to perturb the membrane permeability of the phospholipid bilayer was measured by following the leakage of the fluorophore 5-carboxyfluorescein (CF) (Sigma-Aldrich) induced in CF-encapsulated LUVs (large unilamellar vesicles). Lipid films were prepared as described above at a final concentration of 5 mM. The resulting samples were then dried under vacuum for at least 3 h before hydration and suspension in CF solution at a self-quenching concentration (25 mM) in 10 mM HEPES, 100 mM NaCl, pH 7.2, followed by vigorous vortexing.

To form LUVs, the MLV dispersion was passed 11 times through a mini extruder (Avanti Polar Lipids Inc., Alabaster, AL, USA) equipped with two stacked 1 μm polycarbonate filters (Avanti, Alabaster, AL). Free CF was separated from the CF-containing LUVs using the size exclusion chromatography column Superdex 30 Increase 10/300 GL (GE Healthcare, Milan, Italy) connected to an AKTA FPLC system (GE Healthcare, Milan, Italy) by elution with 10 mM HEPES, pH 7.2, containing 100 mM NaCl. For the assay, CF-entrapped liposomes were incubated with RKW at 30 μM concentration in 10 mM HEPES, 100 mM NaCl, pH 7.2, for 30 min at 25 °C. Data were collected every 3 min using an excitation wavelength of 490 nm and an emission wavelength ranging from 500 to 600 nm. At the end of each experiment, complete leakage (100%) of LUVs was achieved by adding the detergent Triton X-100 (0.25%) to the samples. The percentage of CF release was calculated according to the following equation

2 $$\%\;CF\;leakage=\left(F_{t}-F_{i}\right)\times 100/\left(F_{d}-F_{i}\right)$$

where *F*_t_ and *F*_i_ are the fluorescence intensity in the presence and absence of peptide, respectively, and *F*_d_ is the fluorescence measured after final disruption with Triton X-100. All experiments were performed in triplicate.

### 2.7 Circular Dichroism

CD spectra were recorded on a Jasco J-815 CD spectrophotometer (JASCO, Tokyo, Japan) using a 0.1 cm quartz cuvette with a path length of 0.1 cm. Far-UV spectra were recorded from 195 to 250 nm with a 1 nm bandwidth at 25 °C, and a scan speed of 20 nm/min. Peptide solutions of 50 μM in HEPES 10 mM, NaCl 100 mM, pH 7.2, in the absence or presence of 750 μM SUVs were subjected to CD measurements up to 6 h. Results are expressed in terms of mean residue molar ellipticity [θ] (degrees cm^2^ dmol^–1^). The final spectra were obtained by averaging three measurements after subtracting the baseline signal from either the buffer or lipid solutions.

### 2.8 Morphological Characterization and Surface Charge Measurements

The hydrodynamic size and ζ-potential of *Salmonella*-like, *Pseudomonas*-like, and *Staphylococcus*-like liposomes were measured by Zetasizer Nano-ZS instrument (Malvern Instrument Ltd., Cambridge, United Kingdom) equipped with a He–Ne laser (633 nm, fixed scattering angle of 173°, 25 °C). The average size (*d*) of the obtained liposomes (at an initial concentration of 2 mM) was measured by diluting them down to 0.2 mM in Milli-Q water. The liposome suspensions (1 mL) were inserted in a standard disposable cuvette, and three measurements (*n* = 3) of their size were performed. The hydrodynamic size measurements were also performed after 30 min interaction with RKW peptide (0.3, 0.6, 1.5, 3, 6, and 12 μM). The ζ-potential of the bacterial-mimic liposomes and the peptide (before and after their interaction with them) was measured in triplicate (*n* = 3) by using disposable zeta-potential cuvettes (1 mL). ζ-Potential measurements were performed for liposome/peptide interaction monitoring in suspensions of *Salmonella*-like, *Pseudomonas*-like, and *Staphylococcus*-like liposomes (0.3, 0.6, 1.5, 3, 6, and 12 μM). Analogously, a control with eukaryotic-like liposomes was performed by measuring DLS and ζ-potential before and after incubations with RKW at different concentrations (0.3, 0.6, 1.5, 3, 6, and 12 μM).

### 2.9 Toxicity Assay

*Caenorhabditis elegans* strain N2 was grown on nematode growth medium (NGM) agar plates at 20 °C and fed with *Escherichia coli* OP50.^21^ Adult nematodes were transferred to 96-well plates (20 worms/well), each well containing 50 μL of S medium supplemented with *E. coli* OP50, according to the method of Stiernagel.^21^ Peptide was added to the wells at concentrations of 0, 10, and 50 μM. To assess worm survival after 48 h of peptide treatment at 20 °C, the plate was manually shaken in the multiwell and the worms were considered dead if they did not move and appeared as rigid rods.^22^

### 2.10 Screening in *C. elegans*

Young adult hermaphrodite *C. elegans* worms, grown at 20 °C on NGM agar plates inoculated with *E. coli* OP50, were individually transferred to fresh NGM plates containing peptide RKW at 0, 10, and 50 μM concentrations and seeded with *E. coli* OP50 supplemented with the corresponding peptide RKW concentrations. Worms were incubated at 20 °C and transferred to fresh identical plates every 24 h until all fertilized eggs were laid (3 days).

Embryonic survival was assessed by scoring the eggs 24 h after laying; the ratio of hatched eggs to total eggs laid was calculated as a measure of survival. The total count of eggs laid by a single worm over 3 days. Developmental defects were monitored for up to 96 h postlaying.^23^

### 2.11 Quantitative Analysis of Germline Apoptosis

Adult nematodes exposed to peptide RKW 0, 10, and 50 μM concentrations throughout their development, from egg laying to the adult stage, were suspended in M9 solution and stained by incubation with 33 μM SYTO-12 (Molecular Probes) for 1 h and 30 min at 20 °C in the dark. The worms were then transferred to seeded plates to allow stained bacteria OP50 to be purged from the gut. After 30 min, the animals were mounted on 2% agarose pads in 2 mM levamisole. Quantitative analysis was performed using a Leica DM6 fluorescence microscope. Estimation of apoptotic levels for each treatment was calculated as the average number of apoptotic nuclei per gonadal arm.^24^

### 2.12 Statistical Analyses

Statistical analyses were performed using GraphPad Prism, version 8.0.1 (GraphPad, San Diego, CA, USA). All experiments were performed at least three times, and the data were presented as the mean (M) ± standard error (SE). GraphPad Prism was used to assess the data from the fluorescence assays (blue shift, quenching and leakage assays).

## 3 Results and Discussion

### 3.1 Binding of RKW to Model Membranes

In a previous study, the antimicrobial activity and structural characteristics of the 13-residue cationic peptide RKW were characterized.^14^ To further elucidate the mechanism underlying its biological effects, the present work extended this investigation using liposomes as membrane-mimetic systems. Liposomes are widely used as model systems to study lipid bilayers because they closely replicate the structural and functional features of biological membranes. They can be classified by size as small (SUVs, 20–100 nm), large (LUVs, >100 nm), and giant unilamellar vesicles (GUVs, >1000 nm) or by multilamellar vesicles (MLVs, >5), oligolamellar vesicles (OLVs, 2–5), and multivesicular vesicles (MVVs, 1).^25^ Among these, single-lipid bilayers have been particularly valuable for elucidating fundamental membrane properties and understanding peptide–lipid interactions.

To examine peptide–membrane interactions, the binding propensity of RKW was assessed by monitoring changes in tryptophan (Trp) fluorescence as a function of the lipid-to-peptide molar ratio, employing multilamellar vesicles (MLVs) of distinct phospholipid compositions (Figure 1). Upon addition of bacterial vesicles up to 1.8 mM, a pronounced increase in fluorescence intensity was observed (Figure 1A–E), accompanied by a significant blue shift in the emission maximum (Figure 1F). These spectral changes indicate that the Trp residues experienced a less polar environment, suggesting their direct involvement in interactions with bilayers containing negatively charged phospholipids and a concomitant restriction of their conformational mobility. In contrast, neither substantial blue shifts nor notable increases in fluorescence intensity were detected in the presence of neutral vesicles, implying that the Trp microenvironment remained largely unaltered in zwitterionic membranes.

**Figure 1:**
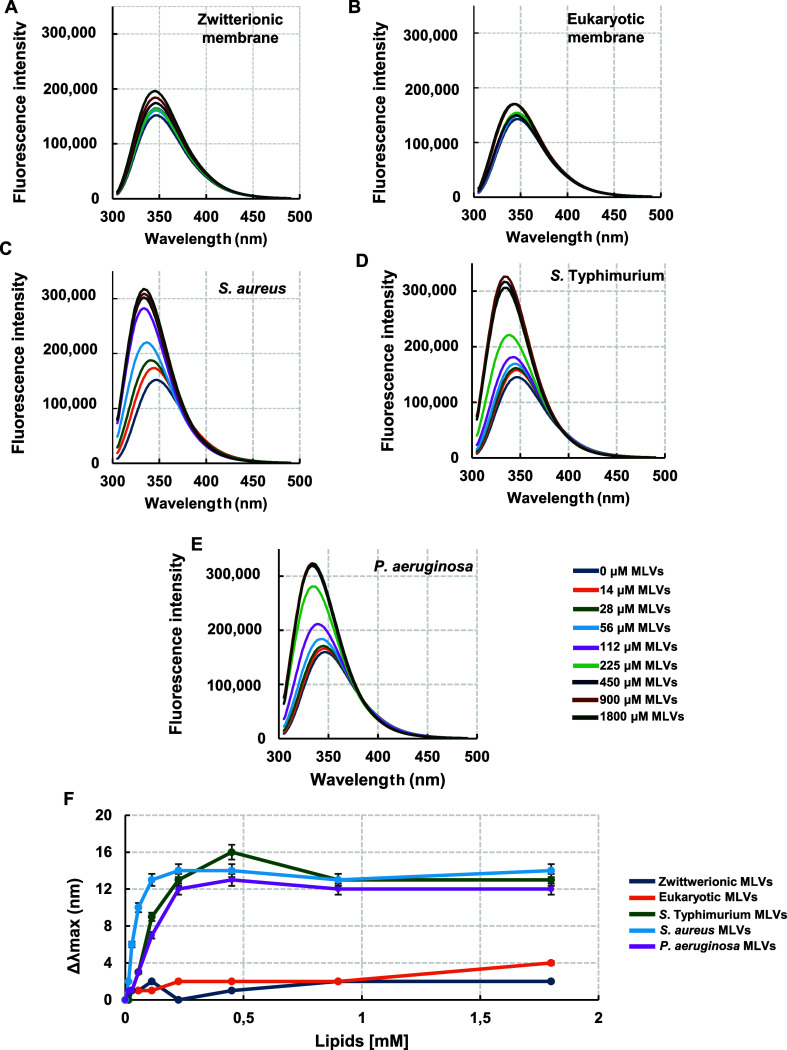
Changes in Trp fluorescence emission spectra of RKW upon interaction with MLVs. Fluorescence emission spectra of RKW in the absence (buffer) or presence of increasing concentrations of (A) zwitterionic, (B) eukaryotic, (C) *S. aureus*, (D) *S. typhimurium*, (E) *P. aeruginosa* multilamellar vesicles (MLVs). (F) Blue shifts in tryptophan fluorescence emission, determined as the difference (Δλ_max_ = λ_0_ – λ) between the wavelengths at the maximum emission in the absence (λ_0_) or presence of lipids (λ), are plotted as a function of increasing lipid concentrations. Measurements were performed in 10 mM HEPES, 100 mM NaCl, pH 7.2 at 25 °C, using a peptide concentration of 20 μM. All data are presented as the mean ± standard deviation (SD). Standard deviation values lower than 5% are not shown.

The observed blue shift correlated well with the binding parameters *K*_d_ (dissociation constant) and Bmax (maximal binding capacity) derived by fitting the binding isotherms (Figure 2A) to a one-site binding model via linear regression (Figure 2B). The results revealed that RKW exhibited lower *K*_d_ and Bmax values toward Gram-negative bacteria (*Salmonella* and *Pseudomonas*) compared to the Gram-positive *Staphylococcus*, with the strongest and most specific interaction observed for *Salmonella*. This behavior likely reflects differences in lipid composition and membrane architecture not only between Gram-negative and Gram-positive bacteria but also among Gram-negative species themselves. Furthermore, no significant changes were revealed in binding curves obtained from MLVs mimicking zwitterionic or eukaryotic membranes (Figure 2), indicating that RKW exhibits negligible affinity for uncharged bilayers. Collectively, these findings suggest that the initial interaction of RKW with bacterial membranes is primarily driven by electrostatic forces rather than hydrophobic effects.

**Figure 2:**
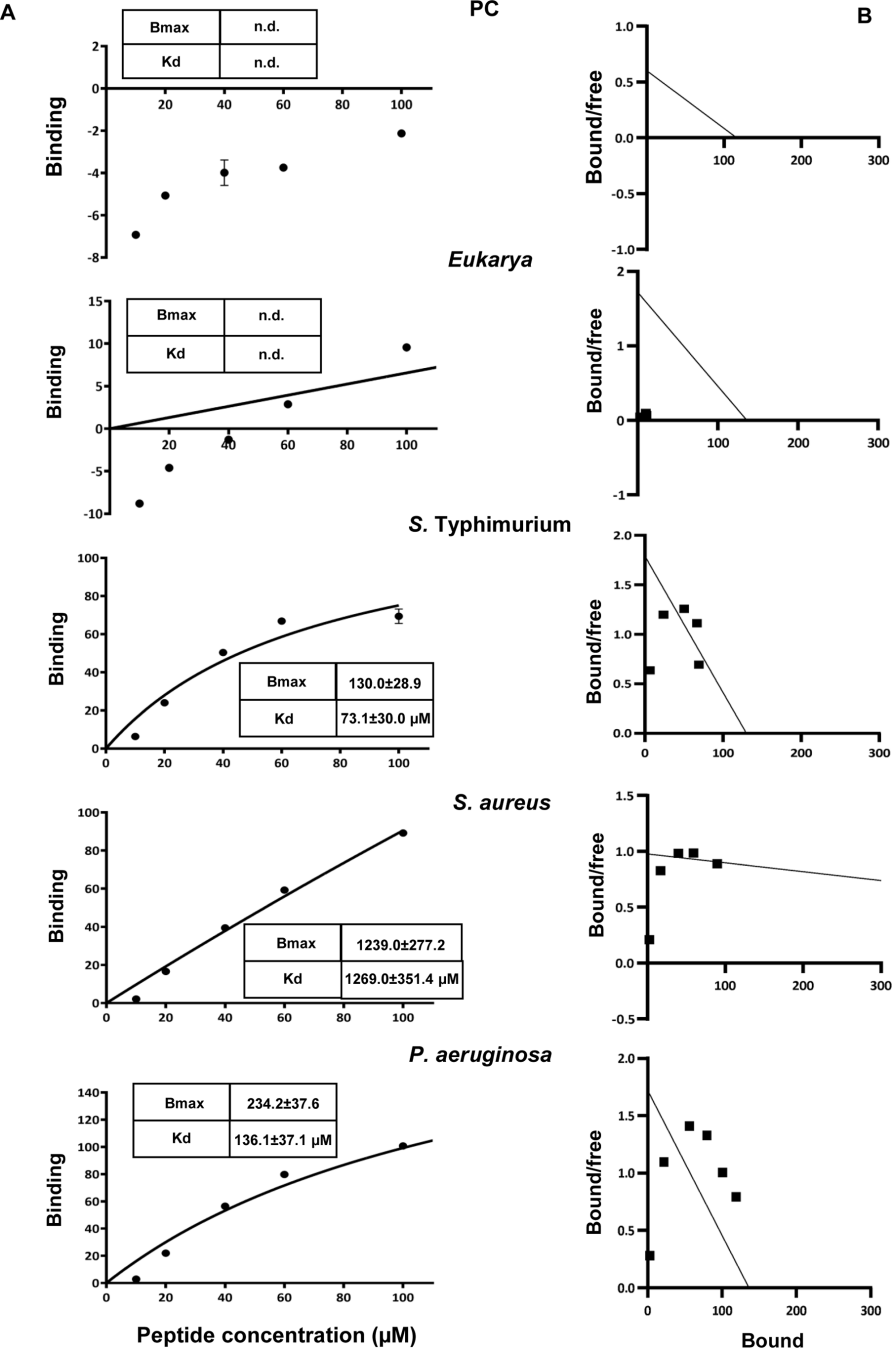
Binding of RKW to MLVs. (A) Binding isotherms calculated from Trp fluorescence intensity at 335 nm for RKW at different concentrations to multilamellar vesicles of varying lipid compositions (1.8 mM) in HEPES buffer and NaCl 150 mM. (B) Scatchard plot analysis for the binding data of RKW to MLVs. All experiments were performed in quadruplicate on different preparations. Data are presented as mean ± standard deviation (SD). Standard deviation values lower than 5% are not shown. n.d: not determined.

### 3.2 Localization of RKW in the Lipid Bilayer

The observed changes in tryptophan fluorescence emission upon RKW binding to bacterial lipid vesicles confirm its interaction with the bilayers. To complement the binding assays and further characterize this interaction, the extent of peptide penetration into the hydrophobic core of the membranes was evaluated by fluorescence quenching using acrylamide, a neutral and hydrophilic quencher of Trp residues. Fluorescence spectra of RKW were recorded at increasing acrylamide concentrations, both in the absence and presence of SUVs with different lipid compositions, at three peptide-to-lipid molar ratios (Figure S1). The corresponding Stern–Volmer plots are shown in Figure 3. In aqueous solution, used as a control for noninternalized peptide, tryptophan fluorescence was quenched efficiently, and in a dose-dependent manner. In contrast, a pronounced decrease in quenching efficiency was observed when RKW was bound to anionic SUVs, indicating that Trp residues were shielded from the aqueous phase due to insertion into the lipid bilayer, thereby reducing their accessibility to acrylamide.

**Figure 3:**
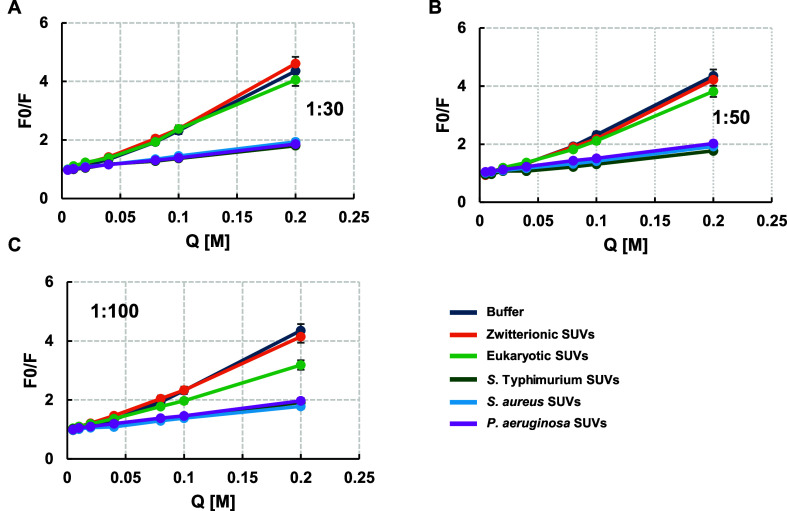
Stern–Volmer plots of RKW quenching by acrylamide. Stern–Volmer plots for the quenching of the Trp fluorescence emission of RKW by acrylamide (*Q*) in aqueous buffer and in the presence of small unilamellar vesicles of varying lipid compositions. The quenching experiments were performed by incubating a 2 μM peptide solution, either in the absence or presence of SUVs at a peptide/lipid (P/L) molar ratio of (A) 1:30, (B) 1:50, (C) 1:100, with increasing acrylamide concentrations ranging from 0 to 200 mM. Spectra were acquired after 30 min of incubation. Experimental data are fittings of the Stern–Volmer eq 1. All values are mean values from at least three independent experiments performed in duplicate. Standard deviation values lower than 5% are not shown.

This protective effect was markedly reduced in the presence of zwitterionic or eukaryotic model membranes, where acrylamide quenched fluorescence similarly to that in aqueous solution. This behavior suggests minimal interaction between RKW and mammalian-like bilayers, leaving Trp residues exposed to the aqueous environment, consistent with the blue-shift observations described earlier (Figure 1). Quantitative analysis of the quenching data yielded Stern–Volmer constants (*K*_sv_) obtained by linear regression (Table 2).

**Table 2:** Stern–Volmer Constants (*K*_sv_) and Normalized Accessibility Obtained from Acrylamide Quenching Studies of Trp Fluorescence of RKW in the Absence or Presence of Differently Charged Vesicles^a^

	buffer	peptide: liposome	zwitterionic membrane	eukaryotic membrane	*S. typhimurium*	*S. aureus*	*P. aeruginosa*
*K*_sv_ (M^–1^)	17.25 ± 1.04	1:30	18.34 ± 2.42	14.49 ± 1.14	4.16 ± 0.24	4.80 ± 0.05	4.44 ± 0.43
		1:50	16.63 ± 4.44	14.20 ± 1.28	4.05 ± 0.55	4.53 ± 0.07	4.98 ± 0.20
		1:100	15.82 ± 0.33	10.87 ± 0.28	4.58 ± 0.04	4.11 ± 0.06	4.85 ± 0.11
NAF	1	1:30	1.06 ± 0.14	0.90 ± 0.07	0.24 ± 0.01	0.28 ± 0.00	0.26 ± 0.02
		1:50	0.96 ± 0.26	0.82 ± 0.07	0.23 ± 0.03	0.26 ± 0.00	0.29 ± 0.01
		1:100	0.92 ± 0.02	0.63 ± 0.02	0.27 ± 0.00	0.24 ± 0.00	0.28 ± 0.00

a NAF is defined in the text, and the *K*_sv_ values were calculated using eq 1.

As expected, *K*_sv_ values decreased nearly 4-fold when transitioning from buffer to negatively charged SUVs, confirming strong peptide–bilayer interactions. Comparable *K*_sv_ values across all bacterial-mimicking membranes indicated a similar degree of tryptophan penetration regardless of specific lipid composition. Furthermore, the Stern–Volmer plots displayed linear behavior in all anionic systems, consistent with a single population of fluorophores equally accessible to the quencher.

Notably, approximately 26% quenching was still observed in the presence of these liposomes, suggesting that Trp residues are not deeply embedded within the hydrophobic core but rather localized near the interfacial region. In contrast, *K*_sv_ values remained largely unchanged in the presence of zwitterionic membranes, indicating limited insertion into these bilayers. These findings were corroborated by the normalized accessibility factor (NAF) values, calculated by normalizing the membrane *K*_sv_ values to those in buffer. NAF values were consistently lower for anionic SUVs than for neutral ones, reinforcing that RKW preferentially inserts into bacterial-like membranes (Table 2).

Because acrylamide is hydrophilic and does not partition into the membrane, this assay cannot distinguish among different depths of peptide insertion. Therefore, to further determine the localization of RKW tryptophan residues within the bilayer, differential quenching experiments were performed using the lipophilic spin probes 16-NS (Figures S2) and 5-NS (Figure S3). These probes contain a doxyl group positioned at distinct depths along the fatty acyl chain, allowing estimation of the peptide’s relative insertion depth. Specifically, 5-NS places the quencher moiety near the membrane–water interface, whereas 16-NS locates it closer to the hydrophobic core of the bilayer.^26,27^ As shown in Figure 4, 5-NS (Table 3) quenched RKW fluorescence more efficiently than 16-NS (Table 4) across all bacterial-mimicking membranes, as reflected by higher *K*_sv_ values. These results indicate that the Trp residues predominantly reside near the bilayer surface, adopting a parallel orientation to the membrane plane and occupying the interfacial region, in agreement with the acrylamide quenching data.

**Figure 4:**
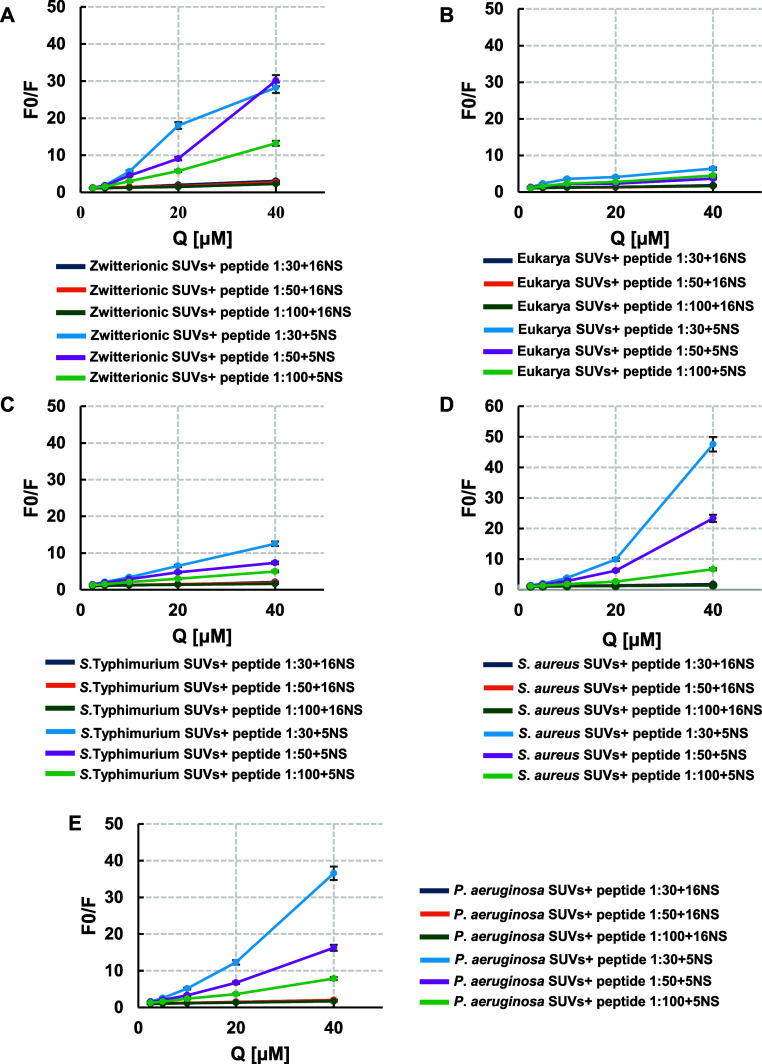
Stern–Volmer plots of RKW quenching by lipophilic probes. Stern–Volmer plots for the quenching of the Trp fluorescence emission of RKW (2 μM) by 16-NS or 5-NS in the presence of small unilamellar vesicles (SUVs) at three different peptide: liposome ratios (1:30, 1:50, 1:100): (A) zwitterionic, (B) eukaryotic, (C) *S. typhimurium*, (D) *S. aureus*, (E) *P. aeruginosa*. Spectra were acquired after 30 min of incubation. Experimental data are fittings of the Stern–Volmer eq 1. All values are mean values from at least three independent experiments performed in duplicate. Standard deviation values lower than 5% are not shown. *Q*: quencher.

**Table 3:** Stern–Volmer Constants (*K*_sv_) Obtained from 5-NS Quenching Studies of Trp Fluorescence of RKW in the Presence of Differently Charged Vesicles, Using eq 1 ^a^

	peptide/liposome	zwitterionic membrane	eukaryotic membrane	*S. typhimurium*	*S. aureus*	*P. aeruginosa*
*K*_sv_ (mM^–1^)	1:30	758.80 ± 50.49	123.20 ± 9.19	297.00 ± 18.53	1235.40 ± 259.65	940.40 ± 46.46
	1:50	772.30 ± 58.05	57.80 ± 0.64	157.80 ± 45.26	590.40 ± 8.84	397.10 ± 47.59
	1:100	324.40 ± 16.97	84.70 ± 0.85	99.00 ± 6.01	146.60 ± 10.32	175.20 ± 8.91

a Data are presented as the best-fit value from three independent experiments ±SD.

**Table 4:** Stern–Volmer Constants (*K*_sv_) Obtained from 16-NS Quenching Studies of Trp Fluorescence of RKW in the Presence of Differently Charged Vesicles, Using eq 1 ^a^

	peptide/liposome	zwitterionic membrane	eukaryotic membrane	*S. typhimurium*	*S. aureus*	*P. aeruginosa*
*K*_sv_ (mM^–1^)	1:30	53.20 ± 4.52	19.20 ± 2.33	29.00 ± 2.61	19.90 ± 1.20	28.20 ± 5.23
	1:50	41.60 ± 10.68	14.20 ± 1.27	22.00 ± 0.07	11.90 ± 0.64	24.40 ± 3.96
	1:100	30.80 ± 2.33	15.40 ± 1.00	14.00 ± 0.07	9.90 ± 0.07	17.00 ± 0.71

a Data are presented as the best-fit value from three independent experiments ±SD.

### 3.3 Dye Leakage Assays and Interaction of RKW with Liposomes

Many AMPs exert their biological activity through mechanisms that involve microbial membrane permeabilisation.^28,29^

Therefore, the ability of RKW to induce membrane damage was evaluated by measuring the time course release of the fluorescent dye 5-carboxyfluorescein (CF) from LUVs of defined compositions. In this experiment, if the peptide induces a bilayer perturbation, a measurable increase in CF fluorescence should be observed due to the leakage of the probe from vesicles. Otherwise, a low fluorescence signal corresponding to 0% leakage is recorded, as expected for the self-quenched and highly concentrated CF entrapped within intact LUVs. The extent of CF release from biomimetic vesicles over time, elicited by the exposure to the peptide, is reported in Figure 5.

**Figure 5:**
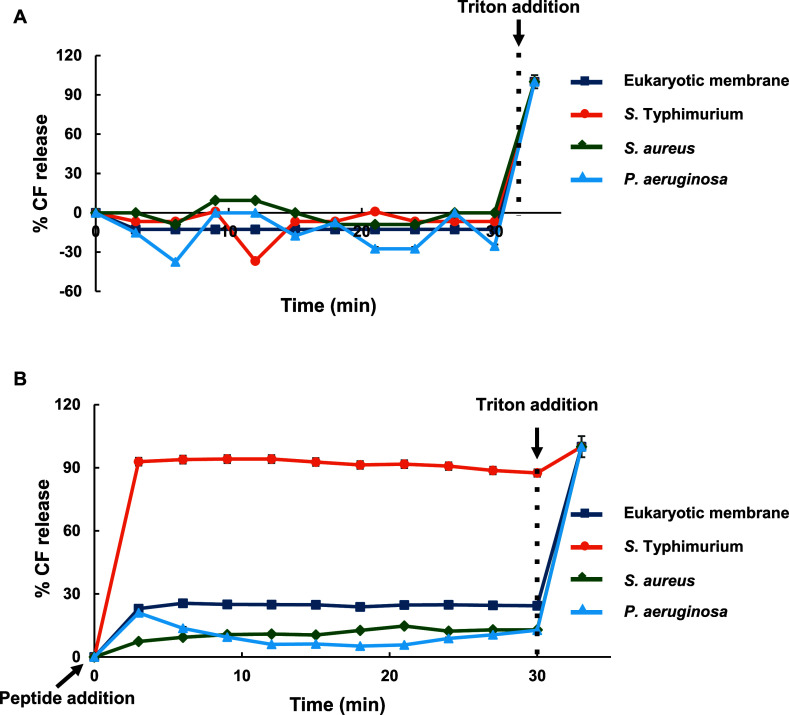
Effect of RKW on LUVs permeability. Leakage of 5-carboxyluorescein from LUVs of varying lipid composition after exposure to RKW. LUVs containing 25 mM CF were prepared by extrusion, using a polycarbonate porous membrane. After 30 min, Triton X-100 (0.25%) was added to induce complete leakage of CF. Fluorescence spectra were measured upon excitation at λ = 488 nm. Leakage is expressed as a percentage relative to the total amount of dye released by the addition of Triton X-100, which represented 100% leakage. The experiments were performed in 10 mM HEPES, 100 mM NaCl, pH 7.2, for 30 min at 25 °C in the (A) absence or (B) presence of RKW. Standard deviation values lower than 5% are not shown. The percentage of CF release was calculated following the eq 2.

In the absence of peptide, a fluorescence intensity corresponding to 0% leakage was observed, reflecting the self-quenched state of the highly concentrated CF entrapped within the inner volume of LUVs (Figure 5A). Upon the addition of RKW, changes in the fluorescence signals were detected.

The leakage kinetics monitored with *Salmonella* vesicles (75% DOPE, 18% POPG, 4% CL) were biphasic, characterized by a rapid initial efflux of CF within the first 3 min, followed by a much slower phase until no further leakage occurred (Figure 5B). In contrast, RKW had a minimal impact on the membrane integrity of the other two bacterial membrane models, similar to the response observed with eukaryotic membranes, resulting in only a limited release of CF (Figure 5B). This suggests that the peptide is less effective at permeabilising membranes resembling *Staphylococcus* (58% POPG, 42% CL) and *Pseudomonas* (60% POPE, 21% POPG, 11% CL) than those mimicking *Salmonella*, consistent with differences in membrane stability and lipid packing. Specifically, the enhanced susceptibility of *Salmonella*-like membranes could be attributed to their high DOPE content, which increases the intrinsic negative curvature stress and lowers the energetic barrier for peptide-induced membrane remodelling, facilitating rapid permeabilization. Conversely, POPG and cardiolipin promote stronger lipid–lipid interactions and local membrane ordering, thereby increasing bilayer cohesion and reducing the likelihood of forming stable permeabilizing defects.^18,30^

Hence, based on the present findings, two considerations can be made from our experimental conditions:

i the peptide could act through a pore-forming mechanism in *Salmonella* membranes, as previously proposed;^31–35^

ii no correlation could be established between the biocidal activity and the extent of membrane disruption caused by RKW.^14^ Since alterations in the functional characteristics of lipid bilayers may only partially explain the lethal action, interactions with other bacterial targets could play a crucial role. Therefore, depending on the different composition of the phospholipid bilayers and the vesicle-peptide ratio, the antimicrobial effect of RKW may result from multiple processes.

### 3.4 Dynamic Light Scattering and ζ-Potential Analysis

To evaluate the effects of RKW on the overall structure of different bacterial membranes and to compare its activity with that on Eukaryote-like membranes, dynamic light scattering (DLS) and ζ-potential measurements were performed. DLS results for all membrane models are shown in Figure 6A. *Salmonella*-like liposomes (0.2 mM) displayed a size distribution with a mean size (d) of 250 ± 50 nm and a polydispersity index—PDI (which is used in the field of light scattering to describe the breadth of a particle size distribution) of 0.3. At low RKW concentrations (0.3, 0.6, and 1.5 μM), the mean size remained unchanged, whereas the PDI increased up to 0.5, indicating partial destabilization of the vesicle population. At higher RKW concentrations (3, 6, and 12 μM), extensive flocculation occurred after 30 min incubation due to membrane disruption and aggregation, resulting in an average size of 3000 ± 800 nm and a PDI of 1.0 (12 μM RKW).

**Figure 6:**
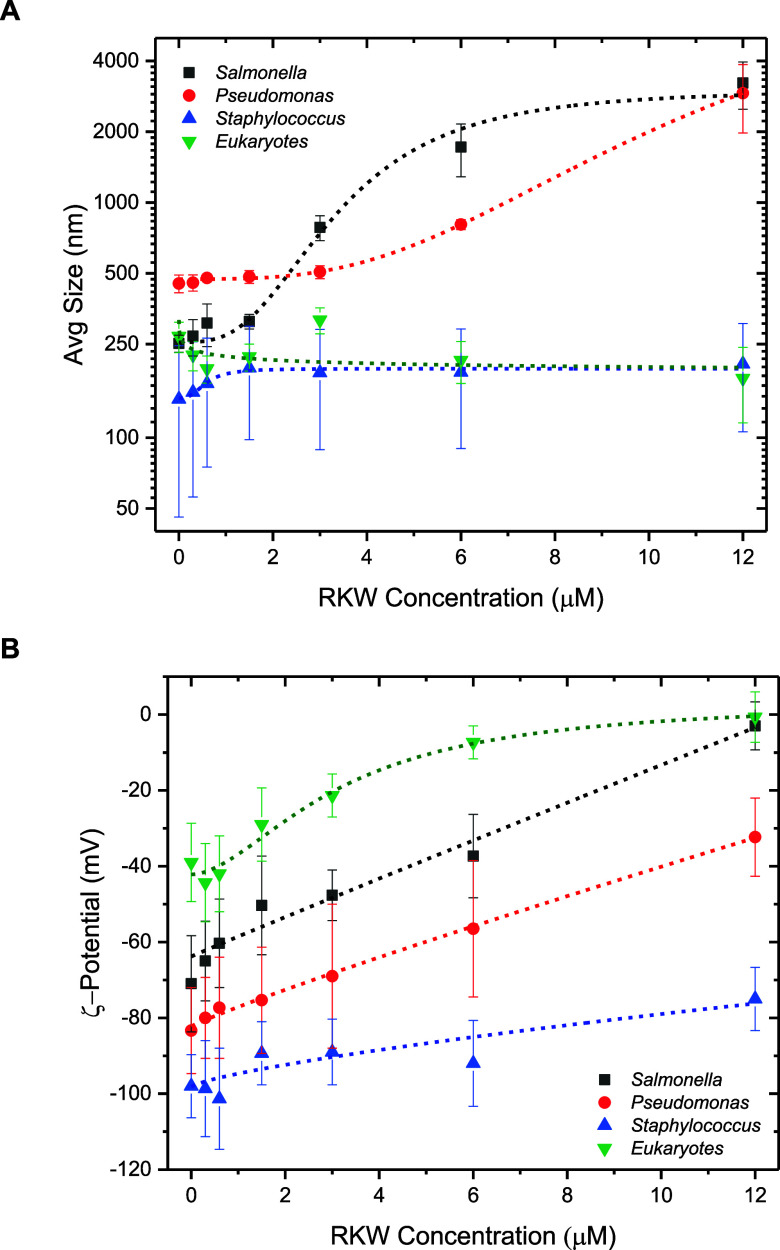
Dynamic light scattering and ζ-potential analysis of bacterial membrane liposomes. (A) Hydrodynamic mean size distribution of *Salmonella*-like liposomes (0.2 mM) (black squares), *Pseudomonas*-like liposomes (0.2 mM) (red circles), *Staphylococcus*-like liposomes (0.2 mM), and eukaryotic-like liposomes (0.2 mM) interacting with RKW (0.3, 0.6, 1.5, 3, 6, and 12 μM). (B) ζ-Potential mean values of *Salmonella*-like liposomes (0.2 mM) (black squares), *Pseudomonas*-like liposomes (0.2 mM) (red circles), *Staphylococcus*-like liposomes (0.2 mM), and eukaryotic-like liposomes (0.2 mM) interacting with RKW (0.3, 0.6, 1.5, 3, 6, and 12 μM). All measures were acquired after 30 min of incubation. Vertical bars denote standard deviation on a minimum of three replicates (*n* ≥ 3). Short dashed lines represent fitted data with logistic curves, illustrating the trend of the measured parameter as a function of RKW concentration.

*Pseudomonas*-like liposomes (0.2 mM) initially exhibited a mean diameter of 400 ± 100 nm and a PDI of 0.4. In contrast to the *Salmonella*-like membranes, a significant size increase was observed only at RKW concentrations ≥6 μM. Below this threshold, the mean size remained constant, while the PDI increased moderately up to 0.6. Upon interaction for 30 min with RKW at 6 and 12 μM concentrations, flocculation of the vesicles was observed due to membrane disruption and aggregation, leading to a size distribution of 3000 ± 700 nm, and an increase in the PDI up to 1.0 (12 μM of RKW peptide). Finally, *Staphylococcus*-like liposomes (0.2 mM) showed a size distribution with a mean size of 150 ± 70 nm and a PDI of 0.6. Across the entire RKW concentration range tested (0.3–12 μM), no significant variations in either size or PDI were detected, suggesting minimal interaction of RKW with this type of membrane under the experimental conditions used.

Overall, these results indicate that RKW exhibits strong activity toward *Salmonella*-like membranes (threshold ≈ 1.5 μM), good activity toward *Pseudomonas*-like membranes (threshold ≈ 6.0 μM), and poor activity toward *Staphylococcus*-like membranes, which would require much higher peptide concentrations to induce structural changes. As expected, Eukaryote-like membranes used as a control (0.2 mM, mean size 250 ± 100 nm; PDI 0.75) did not show any significant variation in mean size as a function of RKW concentration.

ζ-Potential measurements further confirmed these findings (Figure 6B). *Salmonella*-like membranes, initially characterized by a strongly negative surface charge (−70 ± 6 mV), were almost completely neutralized by RKW at 12 μM (−3 ± 5 mV), following a nearly linear trend of surface charge as a function of peptide concentration. A similar behavior was observed for *Pseudomonas*-like liposomes (−80 ± 10 mV), which were partially neutralized to −35 ± 10 mV at 12 μM RKW’s concentration.

In contrast, *Staphylococcus*-like membranes (−100 ± 10 mV) were only slightly affected by RKW, retaining a charge of −80 ± 10 mV even at the highest peptide concentration tested (12 μM).

These results corroborate the strong interaction of RKW with *Salmonella*-like membranes, with respect to *Pseudomonas*-like and *Staphylococcus*-like membranes, suggesting a different mechanism of action of the peptide against the three bacterial models.

Finally, the ζ-potential of Eukaryote-like membranes used as control confirmed a limited peptide–membrane interaction. The surface charge shifted from −40 ± 10 mV in the absence of RKW to −8 ± 5 mV at 6 μM, reaching a plateau thereafter. This partial neutralization was not enough to cause membrane rupture or aggregation, consistent with the DLS observations (Figure 6A). Notably, there was no inversion of surface charge from negative to positive values detected at any RKW concentration.

### 3.5 Circular Dichroism

Membrane active peptides often change their secondary structure upon interaction with lipid bilayers.^36^ This dynamic structural transition is frequently a key step in their mechanism of action, enabling them to exert their biological activity, such as disrupting the membrane, inducing pore formation, or facilitating transport. Therefore, the influence of the membrane interactions on the peptide secondary structure of RKW was investigated using circular dichroism (CD) spectroscopy.

In a previous study, CD experiments were performed using SDS as a membrane-mimicking system. The results revealed that the peptide readily adopts an α-helix conformation when interacting with this membranotropic agent.^14^

However, since phospholipid bilayers are generally considered a more physiologically relevant model of cellular membranes than detergent micelles, the secondary structure of RKW was further investigated in various lipid membrane environments. To this aim, SUVs were employed to minimize light scattering issues typically associated with multilamellar vesicles (MLVs).

The CD spectra showed that the peptide adopts a random coil conformation in aqueous solution, as evidenced by the single minimum signal at 200 nm, typical of a disordered or unstructured state. In the presence of zwitterionic or eukaryotic lipids, the peptide’s secondary structure remained largely unchanged, suggesting a limited or no interaction with these lipid environments (Figure 7).

**Figure 7:**
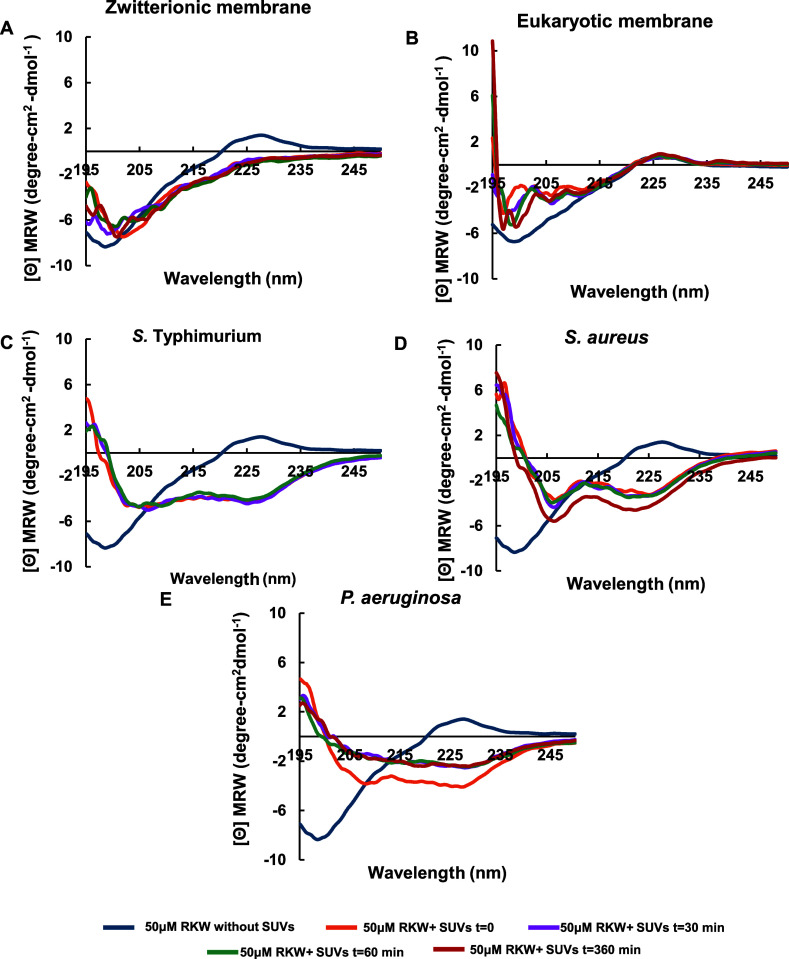
Secondary structure of RKW determined by circular dichroism. Far-UV CD spectra of RKW (50 μM) in the presence of SUVs (750 μM) of varying lipid composition: (A) zwitterionic, (B) eukaryotic, (C) *S. typhimurium*, (D) *S. aureus*, (E) *P. aeruginosa*. The experiments were performed in 10 mM HEPES, 100 mM NaCl, pH 7.2, at 25 °C.

In contrast, a marked conformational change was observed upon the addition of anionic SUVs. Specifically, the CD spectra displayed two distinct and well-defined minima at approximately 208 and 220 nm, along with a maximum near 195 nm, indicative of a conformational transition into a more ordered α-helical structure upon binding to vesicles of different lipid composition, in good agreement with the data obtained in SDS micelles.^14^ Notably, interaction of RKW with *Salmonella*-mimicking membranes led to vesicle aggregation and precipitation, which hindered spectral acquisition beyond 60 min. These results align with those from binding assays previously described, which demonstrated a higher affinity of the peptide for *Salmonella* liposomes compared to other membrane models tested.

### 3.6 In Vivo Studies of RKW on *C. elegans*

In a previous study, the absence of cytotoxicity of RKW was demonstrated in vitro using mouse embryo fibroblasts (BALB 3T3 clone A31), as assessed by the neutral red uptake (NRU) assay in accordance with ISO 10993–5.^14^ Consistent with these findings, and to further support the promising safety profile of RKW, in vivo assays were performed in the present work using *C. elegans* as a model system.

Over the past decade, *C. elegans* has become a widely used organism in biomedical and toxicological research to investigate the effects of peptide exposure and other bioactive compounds, due to its numerous advantageous biological features.^37^ Indeed, *C. elegans* is one of the most well-characterized organisms at the genetic, physiological, molecular, and developmental levels.^38^

In this context, young adult hermaphrodite worms (Figure 8A), grown on a standard bacterial diet (*E. coli* OP50), were individually transferred to wells containing 50 μL of S medium with *E. coli* OP50 and two different concentrations of RKW (10 μM and 50 μM). After 48 h of treatment, the peptide’s impact on worm survival was evaluated. As shown in Figure 8B,C, RKW did not exhibit any toxicity at the tested concentrations.

**Figure 8:**
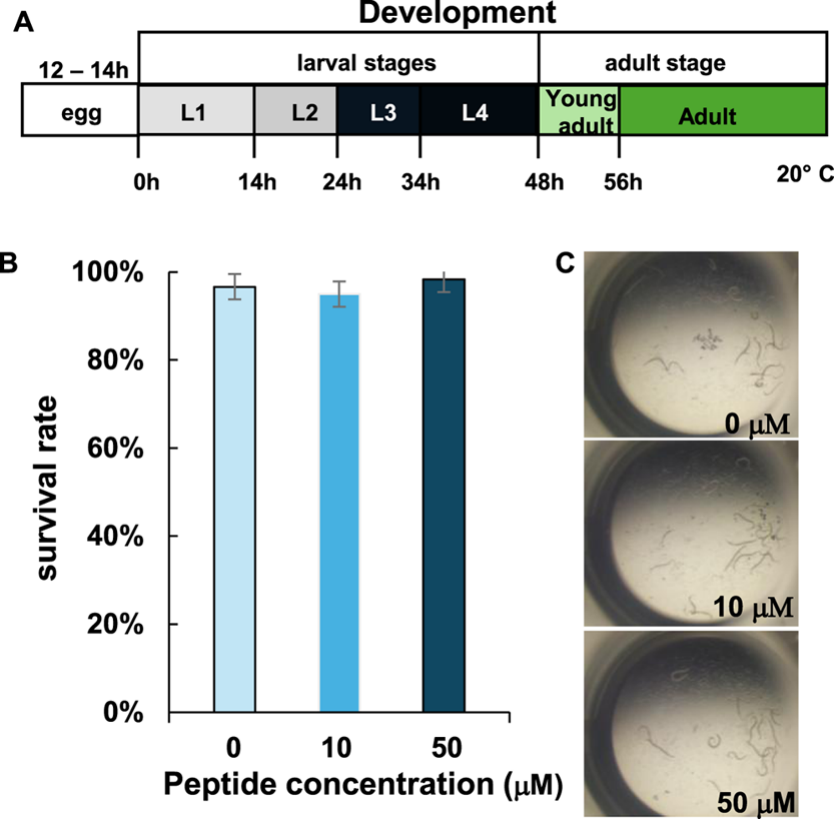
Effects of RKW on *C. elegans* survival. *C. elegans* strain N2 was grown at 20 °C for 48 h in the presence of RKW at two different concentrations. *C. elegans* receiving no treatment served as the control. (A) Schematic representation of the development of *C. elegans*. (B) Percentage of surviving worms after 48 h of treatment with the indicated peptide concentrations. Data represent the mean ± SD of three technical replicates (*n* = 60 worms per condition). (C) Representative images of *C. elegans* worms in wells containing the indicated peptide concentrations after 48 h of treatment. The images were acquired by the author A.A.

Next, to explore the impact of the peptide exposure on progeny development, young adult hermaphrodite worms were transferred to NGM plates containing 10 μM or 50 μM peptide and constantly maintained at 20 °C. Wild-type worms were allowed to lay eggs for 3 days, with parental (P0) worms transferred to fresh plates every 24 h. In this analysis, three parameters were measured: egg-laying rate, embryonic survival and the presence of abnormal phenotypes in the F1 progeny. As shown in Figure 9, treatment with peptide at both 10 and 50 μM concentrations did not significantly affect egg-laying (Figure 9A–C) or embryonic survival (Figure 9B,C) compared to controls. Importantly, all F1 progeny developed into fertile adults, and no growth defects were observed following peptide exposure (Figure 9C,D).

**Figure 9:**
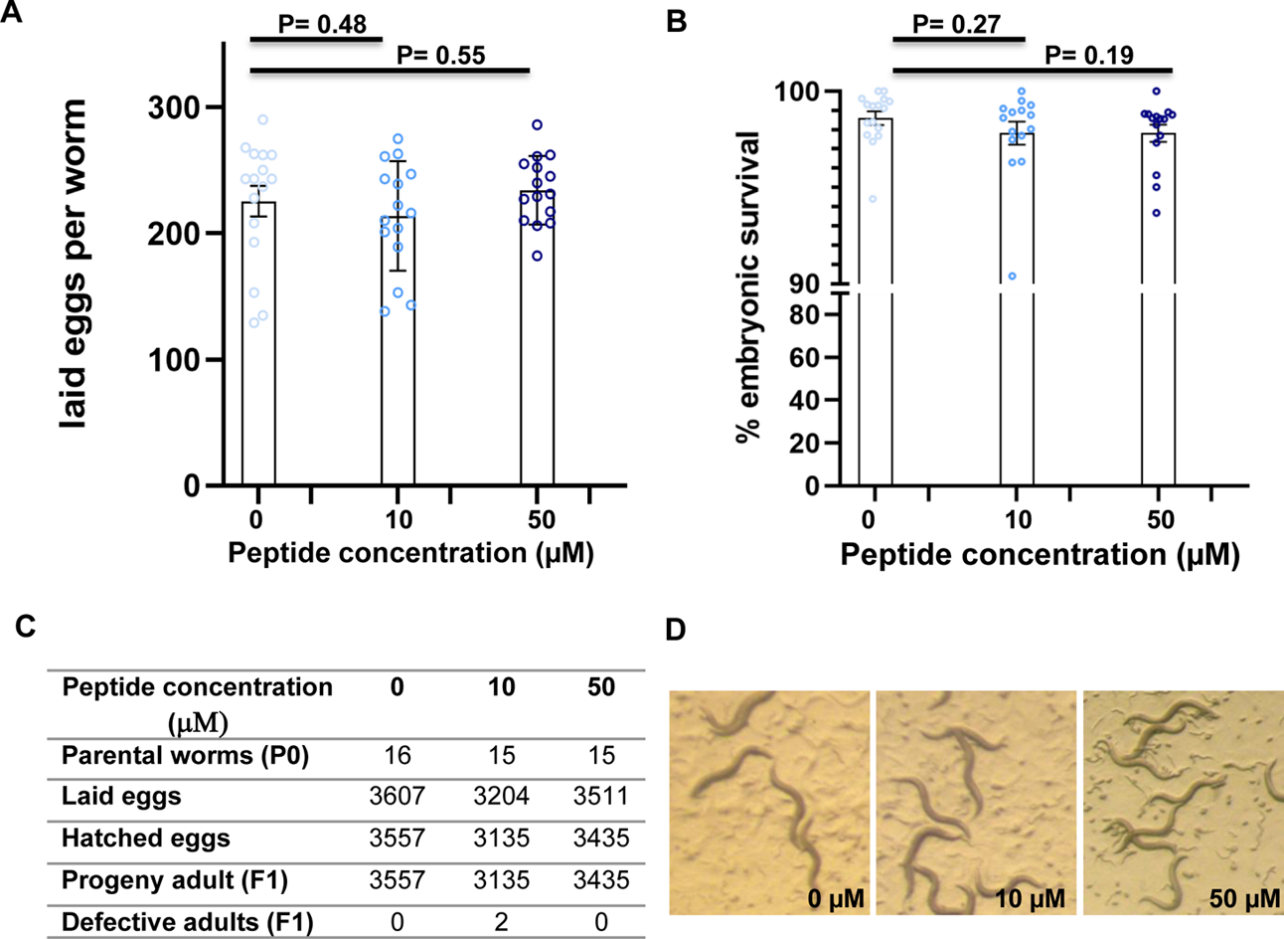
Screening of *C. elegans* worms after treatment with different RKW concentrations. (A) Laid eggs per worm. Bars represent the means ± SEM. Circles represent the number of eggs laid per animal. (B) Embryonic survival (%). Bars represent the means ± SEM. Circles represent the percentage of events per parental worms (P0) animal. (C) Screening F1 progenies of worms at different peptide concentrations. (D) Images of plates containing F1 individuals exposed to the indicated peptide concentration, taken 4 days after hatching. *P*-value obtained by the Student’s *t*-test for independent samples.

In *C. elegans*, basal apoptosis plays a key role in maintaining germline homeostasis.^39^ This physiological process can be monitored in vivo using SYTO-12 staining. Additionally, DNA damage-induced apoptosis of germ cells, mediated by the p53 homologue CEP-1, may occur in response to exogenous DNA damage. This activation triggers the DNA damage checkpoint, leading to increased germline apoptosis, as evidenced by SYTO-12-stained nuclei.^40^ To determine whether RKW exposure could induce genotoxic effects, animals were treated with the peptide throughout their entire development, from egg laying to adulthood, and then stained with SYTO-12. Apoptosis levels were analyzed using a Leica DM6 fluorescence microscope after exposure to RKW at 10 and 50 μM. The results revealed no significant increase in DNA damage-induced apoptosis following peptide exposure (Figure 10).

**Figure 10:**
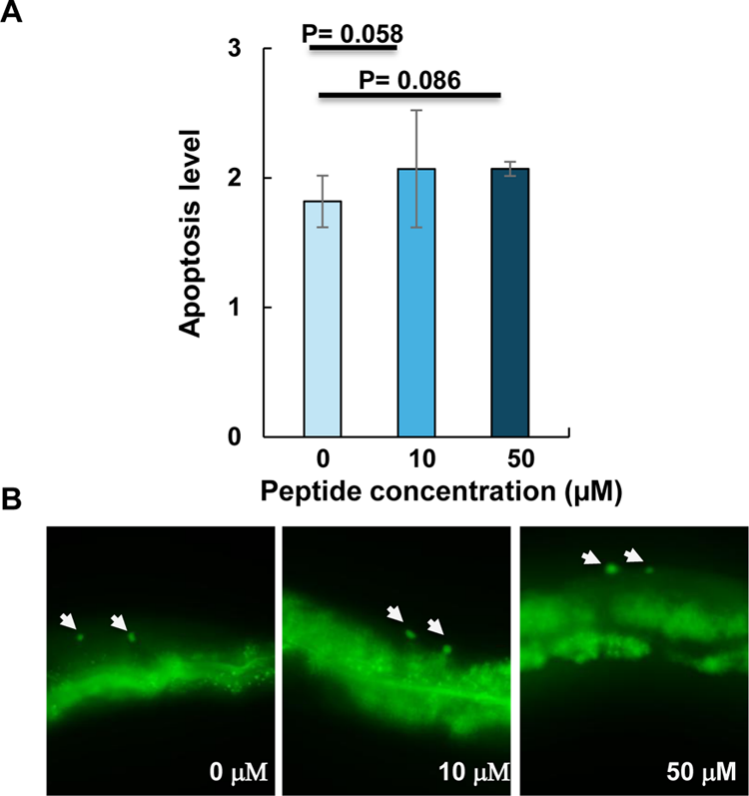
Effect of RKW treatment on germline apoptosis in *C. elegans*. (A) Average apoptosis levels in the germline in the absence (control) or in the presence of RKW at two concentrations. Bars represent the mean ± SEM of three biological replicates. Number of gonads scored: 0:77, 10 μM: 71, 50 μM: 86. *P*-value obtained by Student’s *t*-test for independent samples. (B) Images of SYTO-12 staining of apoptotic cell corpses (arrowheads) in the germline at the indicated treatment.

## 4 Conclusions

Antimicrobial peptides (AMPs) represent a versatile and promising group of bioactive molecules that can be rationally designed to address the growing challenge of antibiotic resistance while minimizing host cell toxicity. Their antimicrobial activity is primarily mediated through interactions with cellular membranes, with permeabilization being one of the most recognized mechanisms of action.^31,33,35,41^ Despite significant progress, the molecular details underlying AMP-induced membrane disruption, specifically in bacterial systems, remain partly understood. To advance our understanding, it is crucial to investigate further the factors that drive the selective interaction of AMPs with biological membranes, considering the complex interplay of physicochemical and environmental factors that influence peptide–lipid interactions. Biophysical techniques and simplified membrane models, such as liposomes, have proven invaluable in this regard, providing essential insights into the mechanisms of peptide action. Moving forward, these approaches will continue to play a pivotal role in uncovering the full potential of AMPs as therapeutic agents.

In this study, the structure and membrane insertion of RKW in zwitterionic and anionic bilayers were determined by using a multidisciplinary approach to gain insight into its mechanism of action. First, the ability of the peptide to bind to the lipid bilayers was monitored by following the change in the fluorescence signal of the intrinsic Trp residues in the peptide chain. Fluorescence titration analyses revealed that RKW interacts strongly with the anionic bilayers, with affinity depending on lipid composition. Further, a marked blue shift in Trp fluorescence upon interaction with bacterial-mimicking membranes indicated that Trp residues become embedded within the hydrophobic core, suggesting effective shielding from the aqueous environment. These findings were in agreement with quenching assays, which demonstrated that RKW resided close to the lipid–water interface region in all three bacterial liposome preparations.

One of the interesting results is the demonstration of two distinct modes of action of RKW against bacterial membranes. Leakage experiments with CF-entrapped liposomes demonstrated that the electrostatically driven interaction between RKW and Salmonella membranes resulted in a significant permeabilisation effect, supporting a lytic mechanism of action of the peptide toward this type of bilayer. Conversely, only minimal membrane damage was detected for the other two bacterial membranes, indicating that a different mechanism underlies its antibacterial activity, which could be strongly dependent on the membrane’s lipid composition, as also confirmed by DLS and ζ-potential analyses. Analysis of the CD spectra revealed that membrane-bound RKW forms a stable α-helical structure in the presence of the bacterial lipid bilayers, most likely induced by selective interactions with the anionic lipids through a combination of electrostatic and hydrophobic effects.

Finally, in vivo studies on the nematode model organism *C. elegans* have demonstrated the absence of toxicity of the peptide, which opens up interesting perspectives for its therapeutic applicability, greatly impacting the search for new drugs able to combat the rising antibiotic resistance infections.

## Supplementary Material



## Data Availability

All data generated or analyzed during this study are included in this article.

## Notes

Figure S1. Fluorescence quenching spectra of RKW by acrylamide; Figure S2. Fluorescence quenching spectra of RKW by 16-NS; Figure S3. Fluorescence quenching spectra of RKW by 5-NS (PDF)
