# Supplementary material for: Selective Interaction of the Antimicrobial Peptide RKW with Bacterial Lipid Bilayers: A Biophysical Approach
Source: ACS Omega. 2026 Feb 11;11(7):12281–95. doi: 10.1021/acsomega.5c11601 (PMC12947016; doi:10.1021/acsomega.5c11601)
Supplement: Supplementary file 1 [file ao5c11601_si_001.pdf]

Selective interaction of the antimicrobial peptide RKW with bacterial lipid bilayers: a biophysical approach

Alessandra Porritiello[a,1], Bruna Agrillo[a,1], Marta Gogliettino[a], Principia Dardano[b], Bruno Miranda[b], Adele Adamo[a], Emanuela Galatola[a], Marco Balestrieri[a] and Gianna Palmieri[a,*]

a *Institute of Biosciences and BioResources - National Research Council (IBBR-CNR), 80131 Napoli, Italy*

b *Institute of Applied Sciences and Intelligent Systems - National Research Council (ISASI-CNR), 80131 Napoli, Italy*

[1] **These authors contributed equally to this work**

Corresponding author.

E-mail address: gianna.palmieri@cnr.it

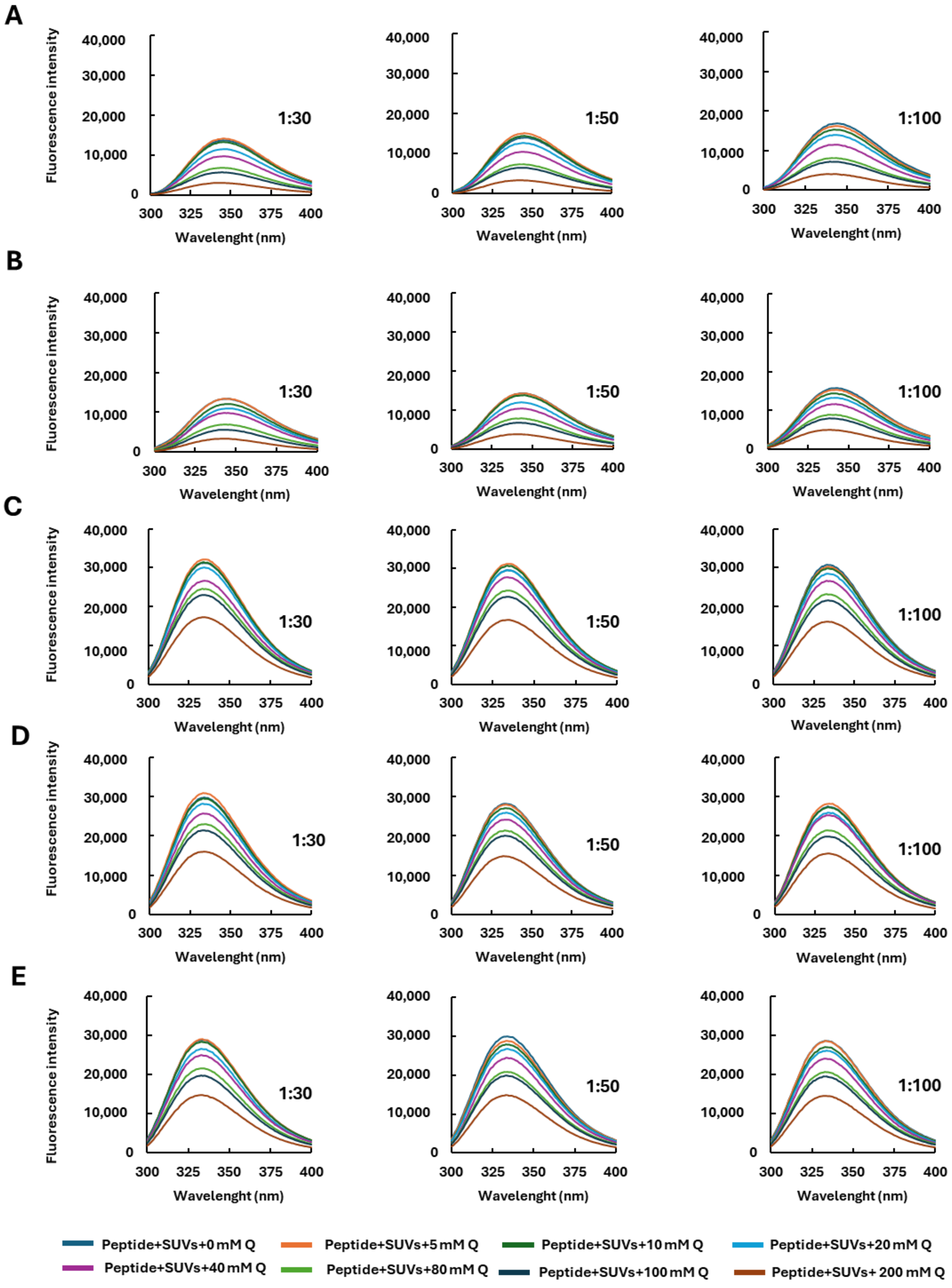

Figure S1. Fluorescence quenching spectra of RKW by acrylamide (Q) in the presence of differently charged small unilamellar vesicles (SUVs) at three peptide/lipid molar ratios (1:30; 1:50; 1:100): (**A**) zwitterionic, (**B**) Eukaryotic, (**C**) *S.* Typhimurium, (**D**) *S. aureus* and (**E**) *P. aeruginosa*. The experiments were performed in 10 mM HEPES, 100 mM NaCl, pH 7.2, at 25 °C after 30 minute incubation.

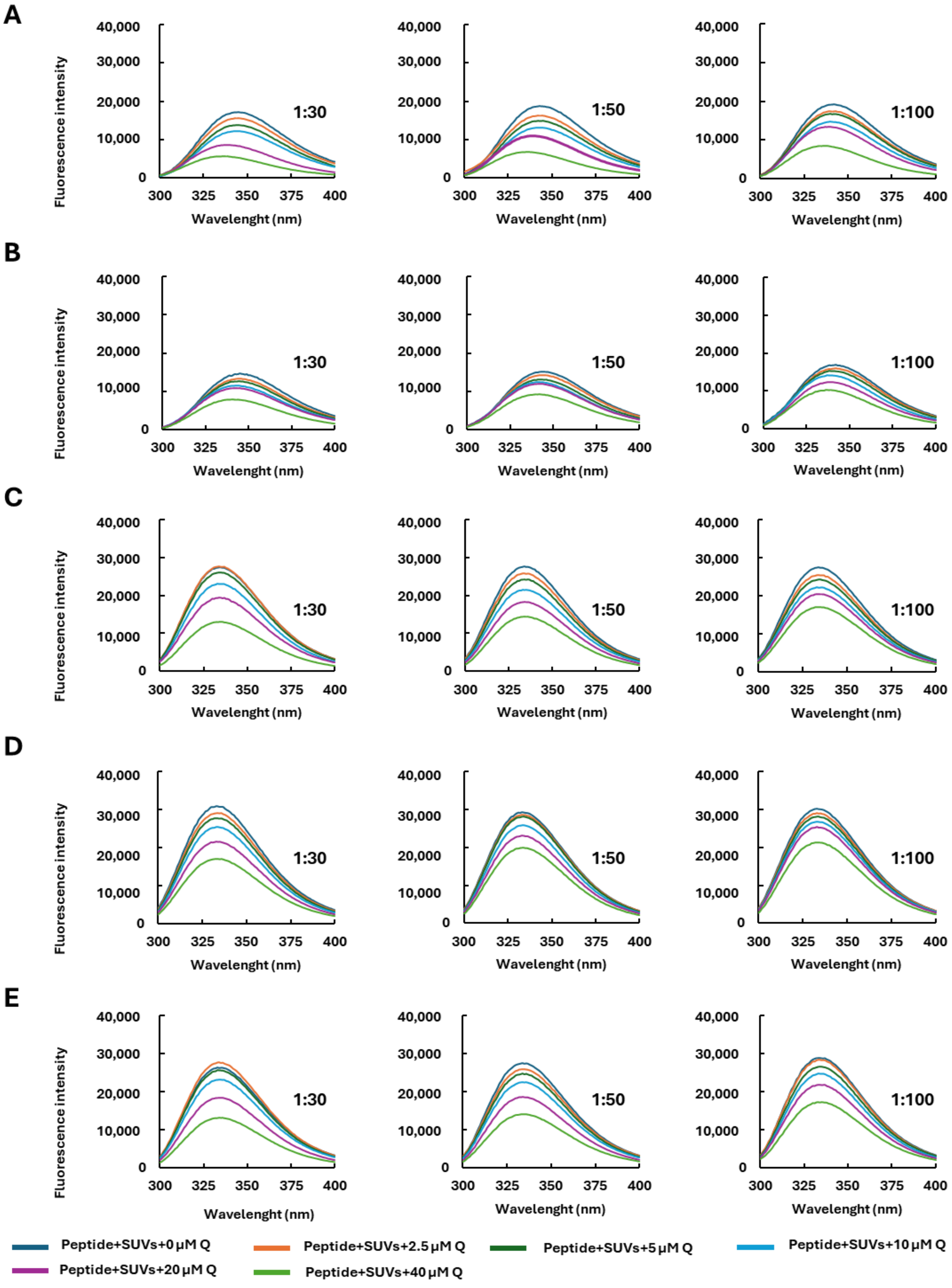

Figure S2. Fluorescence quenching spectra of RKW by 16-NS (Q) in the presence of differently charged small unilamellar vesicles (SUVs) at three peptide/lipid molar ratios (1:30; 1:50; 1:100): (A) zwitterionic, (B) Eukaryotic, (C) S. Typhimurium, (D) S. aureus, and (E) P. aeruginosa. The experiments were conducted in 10 mM HEPES, 100 mM NaCl, pH 7.2, at 25 °C after a 30-minute incubation.

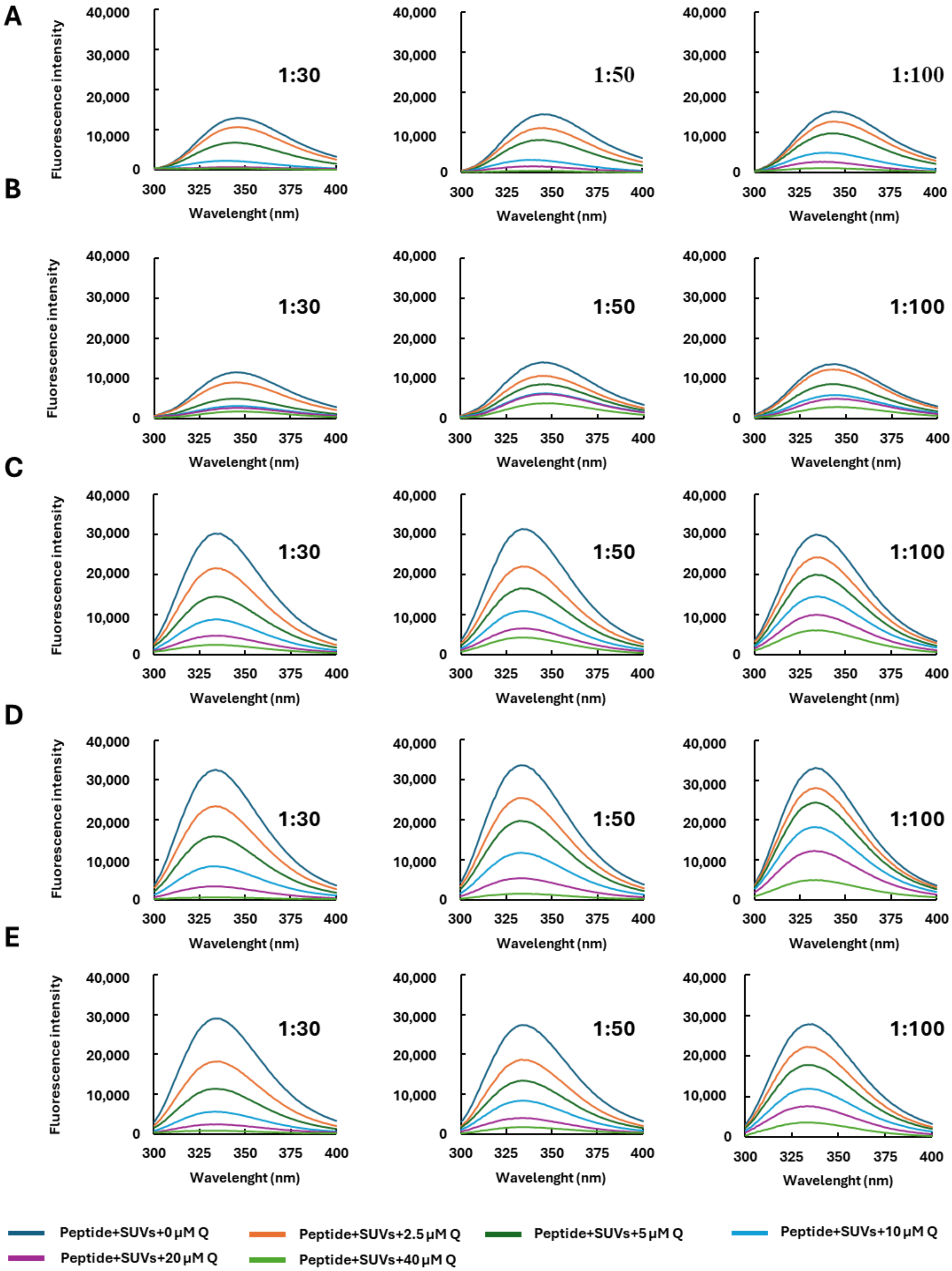

Figure S3. Fluorescence quenching spectra of RKW by 5-NS (Q) in the presence of differently charged small unilamellar vesicles (SUVs) at three peptide/lipid molar ratios (1:30; 1:50; 1:100): (**A**) zwitterionic, (**B**) Eukaryotic, (**C**) *S.* Typhimurium, (**D**) *S. aureus* and (**E**) *P. aeruginosa*. The experiments were performed in 10 mM HEPES, 100 mM NaCl, pH 7.2, at 25 °C after 30 min incubation.
